# Association between vitamin D serum levels and insulin resistance assessed by HOMA-IR among non-diabetic adults in the United States: Results from NHANES 2007–2014

**DOI:** 10.3389/fnut.2022.883904

**Published:** 2022-10-14

**Authors:** Xin Yin, Jia-Yu Chen, Xiang-Jie Huang, Jia-Hong Lai, Chang Huang, Wang Yao, Nan-Xi Li, Wei-Chao Huang, Xu-Guang Guo

**Affiliations:** ^1^Department of Clinical Laboratory Medicine, The Third Affiliated Hospital of Guangzhou Medical University, Guangzhou, China; ^2^Department of Endocrinology, Endocrinology Research Center, Xiangya Hospital of Central South University, Changsha, China; ^3^Department of Clinical Medicine, The Third Clinical School of Guangzhou Medical University, Guangzhou, China; ^4^School of Computer Science and Engineering, Central South University, Changsha, China; ^5^Department of Clinical Medicine, Zhongshan School of Medicine, Sun Yat-sen University, Guangzhou, China; ^6^Department of Psychiatric Medicine, The Mental Health College of Guangzhou Medical University, Guangzhou, China; ^7^Department of Clinical Medicine, The Second Clinical School of Guangzhou Medical University, Guangzhou, China; ^8^Guangdong Provincial Key Laboratory of Major Obstetric Diseases, The Third Affiliated Hospital of Guangzhou Medical University, Guangzhou, China; ^9^Key Laboratory of Reproduction and Genetics of Guangdong Higher Education Institutes, The Third Affiliated Hospital of Guangzhou Medical University, Guangzhou, China; ^10^Guangzhou Key Laboratory for Clinical Rapid Diagnosis and Early Warning of Infectious Diseases, KingMed School of Laboratory Medicine, Guangzhou Medical University, Guangzhou, China

**Keywords:** vitamin D, 25-hydroxyvitamin D, insulin resistance, NHANES, cross-sectional

## Abstract

Insulin resistance, a pathological response to insulin hormone in insulin-dependent cells, is characterized by the presence of high glucose and insulin concentrations. The homeostasis model of insulin resistance (HOMA-IR) is one of the most used indexes to estimate insulin resistance by assessing the fasting glucose and insulin levels. An association was observed between vitamin D levels and insulin resistance, which varied in different ethnic groups, and there is some evidence that vitamin D supplementation could contribute to the improvement of insulin resistance. This study assessed the association between 25-hydroxyvitamin D (25[OH]D) concentration and HOMA-IR in American adults aged 20 years and older, without diabetes and other chronic diseases that can influence insulin resistance. The data from the National Health and Nutrition Examination Survey (NHANES) 2007–2014 were used by exploiting the free and publicly-accessible web datasets. Linear regression models were performed to evaluate the association between serum 25(OH)D concentration and HOMA-IR, and a negative association was observed, which remained significant following the adjustment for age, gender, race/ethnicity, education, body mass index (BMI), physical activity, the season of examination, current smoking, hypertension, the use of drugs which can influence insulin resistance, serum bicarbonates, triglycerides, and calcium and phosphorus levels. Only in non-Hispanic Blacks was this inverse association between vitamin D and HOMA-IR not observed in the fully adjusted model. Further studies are needed to explain the mechanisms of the observed ethnic/racial differences in the association of vitamin D levels with HOMA-IR.

## Introduction

Insulin resistance is identified as an underlying and partly modifiable pathogenic factor of type 2 diabetes mellitus (T2DM) and many related conditions (1). Even though hyperinsulinemic-euglycemic clamp is a gold standard for estimating insulin resistance, it is a quite expensive, invasive, and time-consuming method, which requires trained staff, and therefore, the homeostasis model of insulin resistance (HOMA-IR) presents one of the most simple and suitable substitutes to estimate IR, by assessing the fasting glucose and insulin levels (2).

Vitamin D is the collective name for vitamin D3 (cholecalciferol) and vitamin D2 (ergocalciferol) (3). Surveys from across the globe have shown that vitamin D deficiency was a global health problem that affects people of various ages and nationalities (4, 5). Numerous illnesses, including T2DM (6), obesity (7–9), metabolic syndrome (9, 10), chronic kidney disease (CKD) (11), infective diseases (including COVID-19) (12), autoimmune disorders (13), and infertility (14, 15), have been associated with insufficient vitamin D levels. Many cross-sectional surveys and meta-analyses indicated vitamin D deficiency to be inversely related to HOMA-IR (8, 16), and some meta-analyses (but not all) have shown that supplementation with vitamin D may help control glycemic response and can improve insulin resistance in patients with T2DM (17–19). Additionally, vitamin D receptor (VDR) polymorphisms are associated with insulin resistance and abnormal glucose metabolism, particularly in some ethnic groups (20, 21). Furthermore, a cross-sectional study in the USA, which was performed based on the National Health and Nutrition Examination Survey (NHANES) 2001–2006, found that Non-Hispanic Black people were at a greater risk for insulin resistance compared to White people (22), which may be due to lower serum vitamin D levels.

In this study, we aimed to examine the associations between 25-hydroxyvitamin D (25[OH]D) and HOMA-IR in American adults without diabetes and explore the factors that impact insulin resistance in particular ethnics, using the available data from NHANES 2007–2014, a large-scale and nationally representative cross-sectional surveys of the U.S. population. We hypothesized that the association between insulin resistance and vitamin D would differ across the ethnic groups.

## Materials and methods

### Data source

The National Health and Nutrition Examination Survey is an ongoing, health-related survey that assesses the nutritional and health status of the American population. Survey participants were recruited by a stratified multistage probability sampling method to ensure the sample was nationally representative (23).

The original study protocol was available on the website of the ethics review board of the national center for health statistics research (https://www.cdc.gov/nchs/nhanes/irba98.htm), which was further approved by the ethical review committee (protocol # 2005–06; protocol # 2011–17). The current study was based on the existing data retrieved from NHANES, and the details were extracted from the official website (24).

### Study population

This study used public data retrieved from four cycles of NHANES (2007–2008, 2009–2010, 2011–2012, and 2013–2014). Adult patients aged 20 or older with available data for HOMA-IR and vitamin D were included. The exclusion criteria were the presence of Type 1 diabetes mellitus (T1DM) and T2DM (since in patients with diabetes, HOMA-IR may not be a representative indicator of insulin resistance due to diminished insulin secretion) (25), CKD, and the use of drugs that can influence insulin sensitivity, including antidiabetic drugs, glucose elevating agents, antineoplastics and anti-retroviral agents, adrenal cortical steroids, selective estrogen receptor modulators, parathyroid hormone and analogs, antiandrogens, aromatase inhibitors, calcimimetics, antipsychotics, other metabolic agents, bone resorption inhibitors (bisphosphonates, etc.), and niacin. Although some anti-hypertensive drugs, sex hormones (including contraceptives), and statins can influence insulin sensitivity, the subjects who used those medications were not excluded from the study, because a substantial number of the subjects were using these agents (*N* = 1,081, *N* = 269, and *N* = 561, respectively). Nevertheless, to account for their potential influence on insulin sensitivity, the usage of these drugs was included in covariates in our regression analyses. Participants with any covariates missing were excluded.

Type 2 diabetes mellitus is diagnosed based on plasma glucose levels, including either the fasting plasma glucose value or the 2-h plasma glucose value during a 75 g oral glucose tolerance test or the glycosylated hemoglobin A1c criteria (26). However, either doctor-diagnosed or self-reported diabetes is included for certain. The participants with impaired glucose tolerance or impaired fasting glucose, in case they were not using antidiabetic drugs, were included. CKD was diagnosed based on an increased albumin/creatinine ratio (≥30 mg/g) and a decreased estimated glomerular filtration rate (<60 ml/min/1.73m^2^) (27). The data on the prescription medications were inquired and collected by trained interviewers.

### Measurement

Plasma and serum samples for fasting plasma glucose, serum insulin, 25(OH)D, bicarbonates, total calcium, phosphorus, and triglycerides were obtained and stored in the Mobile Examination Center until shipped to the Centers for Disease Control and Prevention Environmental Health Laboratory (Atlanta, Georgia). The HOMA-IR model was used to evaluate insulin resistance, calculated using the following formula: fasting serum insulin (μU/L) × fasting plasma glucose (mmol/L)/22.5 (28). Concentrations of 25(OH)D3 and 25(OH)D2 in the serum samples were analyzed using super high-ultra performance liquid chromatography-tandem mass spectrometry. Total 25(OH)D (or vitamin D) was defined as the sum of 25(OH)D3 and 25(OH)D2. In terms of the serum total vitamin D levels, the participants were classified as deficient (<50 nmol/L), suboptimal (50–75 nmol/L), and sufficient (>75 nmol/L), as recommended by the American Endocrine Society (29).

### Covariates

We tested all covariates if they were associated with HOMA-IR or vitamin D levels, and the significantly associated covariates were included in the adjusted linear regression models. The eligible covariates included age, gender, race/ethnicity, education, body mass index (BMI), physical activity level (PAL), the season of examination, current smoking, hypertension, the usage of antihypertensive drugs, sex hormones (30, 31), statins (32, 33), serum bicarbonates (34, 35), triglycerides (36, 37), and calcium and phosphorus levels (38–40). The race/ethnicity was divided into five groups: Mexican Americans, Other Hispanics, Non-Hispanic Whites, Non-Hispanic Blacks, and Other races/ethnicities (including Asians and mixes). Education levels were categorized as < 9th grade, 9th−11th grade (including 12th grade with no diploma), high school graduate/general educational development (GED) or equivalent, college/associate of arts (AA) degree, college graduate or above, refused, and unknown. The season of examinations was classified into November to April and May to October. The current smokers were separated from the former and never smokers. Participants who reported smoking either some days or every day at the time of the interview were considered current smokers. Participants who smoked more than 100 cigarettes during their lifetime but did not smoke currently were former smokers. Body mass index (BMI, kg/m^2^) was defined as body weight in kilograms divided by squared body height in meters. Physical activity level (PAL) scores were calculated to assess physical activity based on the different levels of activity, including vigorous (2 points) or moderate (1 point) work-related activity, vigorous (2 points) or moderate (1 point) leisure-time physical activity, and walking or bicycling for transportation (1 point). The minimum PAL score was 0, and the maximum PAL score was 5. Hypertension was defined as having systolic blood pressure ≥ 130 mmHg or diastolic blood pressure ≥ 80 mmHg, which were measured on more than or equal to two occasions to acquire an average (41).

### Statistical analysis

Median and interquartile range (IQR) were used to describe a non-normal distribution. The mean and standard deviation (SD) were used to describe a normal distribution. To compare the differences between various vitamin D status categories, the χ^2^ test (for nominal data), the one-way analysis of variance (ANOVA) (for continuous variables with normal distribution), and the Kruskal-Wallis's test (for continuous variables with non-normal distribution) were used. In linear correlation analyses, any continuous variable that was not normally distributed underwent log 10 transformation to ensure its normal distribution (HOMA-IR, triglycerides). Pearson correlation coefficient (r) was used for normally distributed continuous variables, while Spearman correlation coefficient (r_s_) was used for non-normally distributed continuous variables or ordered categorical variables. The Point-biserial correlation coefficient (r_pb_) was used for dichotomous variables.

The association between total vitamin D and HOMA-IR was evaluated by employing the enter-type linear regression models. Standardized beta was utilized to compare the relative predictive strength of different covariates in the regression models. The variance inflation factor (VIF) was used to assess the multicollinearity of all covariates in the regression model. In linear regression analyses, HOMA-IR and triglycerides underwent log 10 transformation. Stratified regression analyses were used to account for differences between races. Two-tail *p* < 0.05 were considered statistically significant. All analyses were performed using Empower stats (http://www.empowerstats.net/cn/) and SPSS software Version 21.0.

## Results

### Baseline characteristics of participants

**Figure 1:**
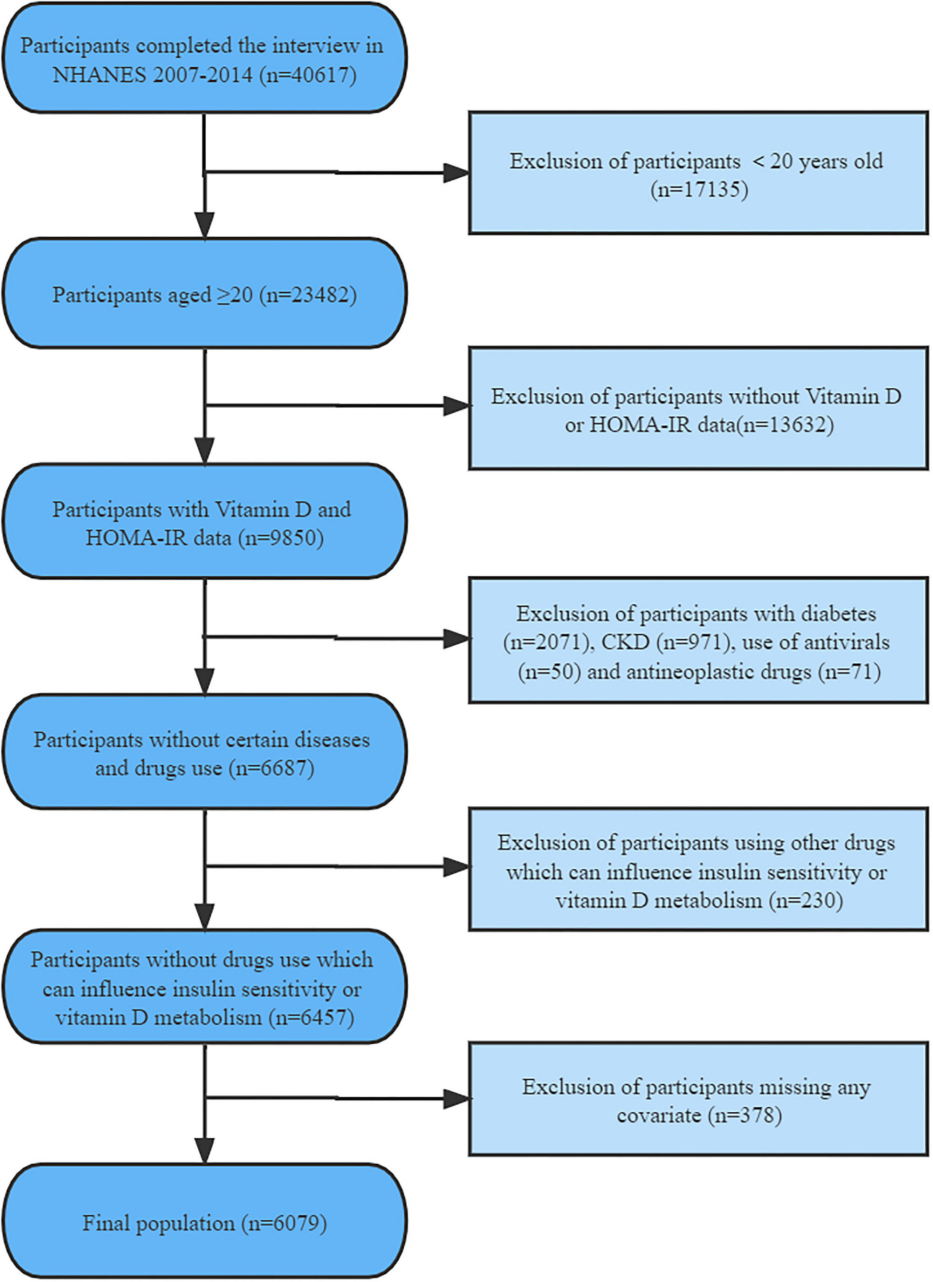
Flowchart of participants' disposition.

Following the exclusions, this study included a total of 6,079 participants aged 20 years or older (Figure 1). Baseline characteristics of the selected participants were classified according to varying serum vitamin D status categories as provided in Table 1.

**Table 1:** Baseline characteristics of participants and distribution across different vitamin D categories.

	**Total** **(*N* = 6,079)**	**Baseline serum vitamin D, nmol/L**			**ANOVA, Kruskal-Wallis's test or χ^2^ test**
		** < 50**	**50-75**	**>75**	***P*** **value**
		**(*N* = 1,968)**	**(*N* = 2,335)**	**(*N* = 1,776)**	
Age, Median (IQR)	43.00 (31.00–56.00)	38.00 (29.00–51.00)	42.00 (31.00–55.00)	48.00 (35.00–60.00)	<0.001
Serum vitamin D (nmol/L), Mean ± SD	63.08 ± 25.88	36.43 ± 9.18	62.27 ± 6.93	93.68 ± 20.29	<0.001
HOMA-IR, Median (IQR)	2.22 (1.40–3.66)	2.57 (1.56–4.23)	2.25 (1.44–3.66)	1.87 (1.20–3.01)	<0.001
Plasma fasting glucose (mmol/L), Mean ± SD	5.42 ± 0.54	5.42 ± 0.54	5.43 ± 0.53	5.39 ± 0.54	0.105
Serum fasting insulin (μU/L), Median (IQR)	9.28 (6.04–14.80)	10.78 (6.66–17.27)	9.45 (6.20–14.66)	7.88 (5.16–12.32)	<0.001
Serum triglycerides (mmol/L), Median (IQR)	1.08 (0.76–1.59)	1.02 (0.71–1.53)	1.14 (0.79–1.65)	1.07 (0.78–1.59)	<0.001
Serum bicarbonates (mmol/L), Mean ± SD	25.21 ± 2.10	25.02 ± 2.07	25.23 ± 2.06	25.39 ± 2.18	<0.001
Serum calcium (mmol/L), Mean ± SD	2.34 ± 0.08	2.33 ± 0.08	2.34 ± 0.08	2.36 ± 0.08	<0.001
Serum phosphorus (mmol/L), Mean ± SD	1.19 ± 0.17	1.19 ± 0.17	1.19 ± 0.17	1.19 ± 0.17	0.290
BMI (kg/m^2^), Mean ± SD	28.10 ± 6.33	29.30 ± 7.32	28.18 ± 5.83	26.66 ± 5.41	<0.001
PAL score, Mean ± SD	1.74 ± 1.30	1.60 ± 1.31	1.76 ± 1.29	1.88 ± 1.29	<0.001
**Gender (** ***N*** **, %)**					<0.001
Men	3,037 (49.96)	962 (31.68)	1,285 (42.31)	790 (26.01)	
Women	3,042 (50.04)	1,006 (33.07)	1,050 (34.52)	986 (32.41)	
**Race/Ethnicity (** ***N*** **, %)**					<0.001
Mexican American	947 (15.58)	394 (41.61)	416 (43.93)	137 (14.47)	
Other Hispanics	667 (10.97)	193 (28.94)	349 (52.32)	125 (18.74)	
Non-Hispanic Whites	2,702 (44.45)	412 (15.25)	1,059 (39.19)	1,231 (45.56)	
Non-Hispanic Blacks	1,102 (18.13)	697 (63.25)	277 (25.14)	128 (11.62)	
Other races	661 (10.87)	272 (41.15)	234 (35.40)	155 (23.45)	
**Season of examination (** ***N*** **, %)**					<0.001
November to April	2,828 (46.52)	1,174 (41.51)	1,061 (37.52)	593 (20.97)	
May to October	3,251 (53.48)	794 (24.42)	1,274 (39.19)	1,183 (36.39)	
**Education (** ***N*** **, %)**					<0.001
< 9th grade	514 (8.46)	180 (35.02)	236 (45.91)	98 (19.07)	
9–11th grade	878 (14.44)	328 (37.36)	338 (38.50)	212 (24.15)	
High school graduate/GED or equivalent	1,309 (21.53)	466 (35.60)	485 (37.05)	358 (27.35)	
College/AA degree	1,782 (29.31)	597 (33.50)	664 (37.26)	521 (29.24)	
College graduate or above	1,589 (26.14)	393 (24.73)	610 (38.39)	586 (36.88)	
Refused or unknown	7 (1.12)	4 (57.14)	2 (28.57)	1 (14.29)	
**Hypertension (** ***N*** **, %)**					0.262
Yes	1,592 (26.19)	541 (33.98)	602 (37.81)	449 (28.20)	
No	4,487 (73.81)	1,427 (31.80)	1,733 (38.62)	1,327 (29.57)	
**Current smoking (** ***N*** **, %)**					<0.001
Yes	1,328 (21.85)	490 (36.90)	487 (36.67)	351 (26.43)	
No	4,751 (78.15)	1,478 (31.11)	1,848 (38.90)	1,425 (29.99)	
**Sex hormones (** ***N*** **, %)**					<0.001
Yes	269 (4.43)	41 (15.24)	69 (25.65)	159 (59.11)	
No	5,810 (95.57)	1,927 (34.92)	2,266 (40.07)	1,617 (29.30)	
**Statins (** ***N*** **, %)**					<0.001
Yes	561 (9.23)	103 (18.36)	203 (36.19)	255 (45.45)	
No	5,518 (90.77)	1,856 (33.64)	2,128 (38.56)	1,534 (27.80)	
**Antihypertensive drugs (** ***N*** **, %)**					<0.001
Yes	1,081 (17.78)	275 (25.44)	398 (36.82)	408 (37.74)	
No	4,998 (82.22)	1,684 (33.69)	1,933 (38.68)	1,381 (27.63)	

AA, associate of arts; ANOVA, analysis of variance; BMI, Body Mass Index; GED, general educational development; HOMA-IR, the homeostasis model of insulin resistance; IQR, inter quartile range; N, number of participants; PAL, physical activity level; SD, standard deviation.

The proportion of vitamin D deficiency did not differ between men and women; however, a higher proportion of vitamin D suboptimal and a lower proportion of vitamin D sufficient was found among men. The highest proportion of vitamin D deficiency was among the Non-Hispanic Blacks (63.25%), followed by Mexican Americans, other races-ethnicities (including Asians and mixes), other Hispanics, and finally, the Non-Hispanic Whites (15.25%). Meanwhile, the highest proportion of vitamin D sufficiency was observed among the non-Hispanic Whites (45.56%), while the lowest was among the non-Hispanic Blacks (11.62%).

As expected, a higher prevalence of vitamin D deficiency was observed in samples collected during the winter period (from November to April). With the exception of those who refused to answer or were left unknown about their education levels, the group with the most vitamin D deficiency belonged to the education groups of 9th−11th grade, whereas the group with the most vitamin D sufficiency was the group of college graduates or above. In the present study population, patients with hypertension and current smokers were more vitamin D deficient. Compared with the vitamin D sufficient subgroup, participants in the deficient subgroup were at the highest HOMA-IR.

Serum vitamin D levels were related to age, sex, race, educational level, BMI, PAL score, the season of examination, smoking status, usage of antihypertensive drugs, sex hormones, and statins, serum fasting insulin, triglycerides, bicarbonates, and calcium levels, as well as HOMA-IR. There was no association found between serum vitamin D levels and plasma fasting glucose, serum phosphorus, and hypertension (Table 1).

### Relationship between serum 25(OH)D and HOMA-IR

Table 2 shows that all covariates, except age and serum calcium, were linearly related to HOMA-IR. Regarding the stratified racial analysis, insulin resistance was found to be different among various races: Mexican Americans and other Hispanics were more prone to higher HOMA-IR, while the Non-Hispanic Whites and other races/ethnicities (including Asiatic) were less susceptible, while the Non-Hispanic Blacks were in the middle (Table 2).

**Table 2:** Screening of covariates based on statistically significant association with log-transformed HOMA-IR.

**Age** ^#b^	0.016	**BMI** ^#a^	0.562^**^
**Gender** ^#c^	−0.033^*^	**PAL score** ^#b^	−0.132^**^
**Race/Ethnicity**		**Season of examination** ^#c^	−0.052^**^
Mexican American^#c^	0.121^**^	**Current smoking** ^#c^	0.047^**^
Other Hispanic^#c^	0.028^*^	**Hypertension** ^#c^	−0.113^**^
Non-Hispanic White^#c^	−0.074^**^	**Antihypertensive drugs** ^#c^	−0.138^**^
Non-Hispanic Black^#c^	0.014	**Sex hormones** ^#c^	0.047^**^
Other races^#c^	−0.069^**^	**Statins** ^#c^	−0.073^**^
**Education** ^#b^	−0.104^**^	**Serum bicarbonate** ^#a^	−0.116^**^
Less than 9th grade^#c^	0.046^**^	**Serum triglycerides (log–transformed)** ^#a^	0.372^**^
9-11th grade^#c^	0.030^*^	**Serum calcium** ^#a^	−0.016
High school graduate/GED or equivalent^#c^	0.029^*^	**Serum phosphorus** ^#a^	−0.091^**^
College/AA degree^#c^	0.017		
College graduate or above^#c^	−0.100^**^		

** ***P*** < 0.01.

* ***P*** < 0.05.

#a Pearson's r.

#b Spearman's r_s_.

#c Point-Biserial's r_pb_.

AA, associate of arts; BMI, Body Mass Index; GED, general educational development; HOMA-IR, the homeostasis model of insulin resistance; PAL, physical activity level.

Linear regression analysis (Table 3) revealed that HOMA-IR was inversely associated with vitamin D levels prior to the adjustments for covariates (Model 1). The unadjusted model described only a small variance in HOMA-IR by using only vitamin D levels (2.8%). Following the adjustments of covariates that included age, gender, specific race/ethnicity, education, BMI, physical activity, the season of examination, current smoking, hypertension, the usage of antihypertensive drugs, sex hormones, and statins, as well as serum bicarbonates, calcium, and phosphorus levels (Model 2), this inverse association between vitamin D and HOMA-IR remained significant, although it decreased. This model explained a much higher variance in HOMA-IR (36.1%). The association of vitamin D with HOMA-IR in the fully adjusted model with added log-transformed triglycerides was even more significant since the standardized regression coefficient for vitamin D increased (Model 3). This model explained the highest percentage of variance in HOMA-IR (41.3%).

**Table 3:** Linear regression relationship for serum vitamin D and log-transformed HOMA-IR in models.

		**Adjusted R^2^**	**Standardized β**	**Non-standardized β (95% CI)**
Vitamin D	Model 1^a^	0.028	−0.168	−0.002 (−0.002, −0.002)^**^
	Model 2^b^	0.361	−0.054	−0.001 (−0.001, 0.000)^**^
	Model 3^c^	0.413	−0.056	−0.001 (−0.001, 0.000)^**^

** *P* < 0.01.

a Model 1 is not adjusted.

b Model 2 adjusted for age, gender, specific race/ethnicity, education, BMI, physical activity, the season of examination, current smoking, hypertension, the usage of antihypertensive drugs, sex hormones, statins, serum bicarbonates, calcium, and phosphorus levels.

c Model 3 adjusted for age, gender, specific race/ethnicity, education, BMI, physical activity, the season of examination, current smoking, hypertension, the usage of antihypertensive drugs, sex hormones, statins, serum bicarbonates, log-transformed triglycerides, calcium, and phosphorus levels.

β, beta (regression coefficients); BMI, Body Mass Index; CI, confidence intervals; HOMA-IR, the homeostasis model of insulin resistance.

In stratified regression analyses (Table 4), only in the Non-Hispanic Blacks, there was no significant inverse association between vitamin D and insulin resistance in the fully adjusted model with serum triglycerides included (Model 3).

**Table 4:** Linear regression relationship for serum vitamin D and log-transformed HOMA-IR in stratification analysis of race/ethnicity.

	**Non-standardized β (95% CI)**		
	**Model 1^a^**	**Model 2^b^**	**Model 3^c^**
Mexican American	−0.003 (−0.004, −0.002)^**^	−0.001 (−0.002, 0.000)^**^	−0.001 (−0.002, 0.000)^**^
Other Hispanics	−0.002 (−0.004, −0.001)^**^	−0.001 (−0.002, 0.000)	−0.001 (−0.002, 0.000) ^¥^
Non-Hispanic Whites	−0.002 (−0.003, −0.002)^**^	−0.000 (−0.001, 0.000)^*^	−0.000 (−0.001, 0.000)^*^
Non-Hispanic Blacks	−0.001 (−0.002, 0.000)^*^	−0.000 (−0.001, 0.000)	−0.000 (−0.001, 0.000)
Other races	−0.002 (−0.003, −0.001)^**^	−0.001 (−0.002, 0.000)^**^	−0.001 (−0.002, −0.001)^**^

** *P* < 0.01.

* *P* < 0.05.

¥ *P* = 0.051.

a Model 1 is not adjusted.

b Model 2 adjusted for age, gender, education, BMI, physical activity, season of examination, current smoking, hypertension, the usage of antihypertensive drugs, sex hormones, statins, as well as serum bicarbonates, calcium, and phosphorus levels.

c Model 3 adjusted for age, gender, education, BMI, physical activity, season of examination, current smoking, hypertension, the usage of antihypertensive drugs, sex hormones, statins, as well as serum bicarbonates, log-transformed triglycerides, calcium, and phosphorus levels.

β, beta (regression coefficients); BMI, Body Mass Index; CI, confidence intervals; HOMA-IR, the homeostasis model of insulin resistance.

In the general population or ethnic subgroups, BMI contributed the most to HOMA-IR, as shown in Supplementary Tables S1, S2. The influence of vitamin D on HOMA-IR was the strongest among other races/ethnicities (including Asiatic) compared to Mexicans and other Hispanics and the Non-Hispanic Whites, while the association was not observed in the Non-Hispanic Blacks (Supplementary Table S2).

## Discussion

The present study confirmed the inverse association between vitamin D and insulin resistance in accordance with many studies in different countries (42–45). However, the direct effect of vitamin D on insulin sensitivity is still controversial, since some meta-analyses indicated that vitamin D supplementation did not have the expected beneficial effects, which could be attributed to suboptimal dosing and short duration of follow-up (46, 47).

The mechanisms by which vitamin D can influence insulin sensitivity are various, and some of them are still unknown. Some studies showed that vitamin D by interacting with VDR in insulin-responsive tissues increased the transcription and number of insulin receptors (48, 49). Also, vitamin D can influence the extracellular calcium concentration and influx through the insulin-responsive cell, subsequently activating the glucose transporters, thus enhancing the response to insulin (50, 51). In addition, vitamin D could block the effect of inflammatory cytokines on insulin signaling by modulating the innate immune system and decreasing inflammatory cytokine secretion (52, 53). It is known that reactive oxygen species (ROS) can trigger insulin resistance (54), while vitamin D accelerates ROS catabolism by enhancing the synthesis of antioxidants and anti-inflammatory cytokines (55). Vitamin D can also modulate insulin sensitivity by activating peroxisome proliferator-activated receptors-δ, which reduces free fatty acid-induced insulin resistance (56, 57). Parathyroid hormone (PTH) can mediate insulin resistance by inhibiting insulin signaling and reducing glucose uptake, while vitamin D could exert an insulin-improving effect by reducing PTH levels (58). Moreover, higher PTH and vitamin D insufficiency can be jointly associated with higher HOMA-IR: the effect of PTH on insulin release from islets depends on vitamin D-related calcium and phosphorus (59, 60).

Regarding racial/ethnic differences in the association of vitamin D with HOMA-IR, one population-based investigation showed that the association between circulating 25(OH)D concentrations and insulin resistance did not differ within race (16). Conversely, other studies demonstrated that vitamin D was inversely associated with fasting insulin and insulin resistance in the Non-Hispanic Whites and Mexican Americans, but not in the Non-Hispanic Blacks (61, 62).

The reason for the lack of this association among the Non-Hispanic Blacks is still not clear. Black people have lower levels of vitamin D and higher levels of PTH compared to White people, so the negative association between vitamin D and insulin resistance should be stronger. However, in the Non-Hispanic Blacks, a decreased sensitivity to the effects of decreased vitamin D and elevated PTH was hypothesized (61, 63). Regarding 25(OH)D clearance, Black people had higher 25(OH)D clearance and lower 25(OH)D levels compared to White people, probably owing to lower levels of vitamin D binding protein (22, 62, 64, 65). The threshold for a sufficient 25(OH)D levels is the lowest among the Non-Hispanic Blacks (44), and the inverse association between 25(OH)D and PTH levels were only observed below a much lower cutoff point for vitamin D in Black people (66–68). As a result, the combined effect of PTH and vitamin D lacks in Black people.

In addition, in one study, it was observed that African Americans had significantly lower triglyceride levels for any given level of insulin sensitivity, compared with other races/ethnicities (69, 70), and in another study, it was observed that low levels of triglyceride could slightly modify the association of 25(OH)D with insulin resistance (71), which probably could explain why there was no significant association between HOMA-IR and vitamin D in the Non-Hispanic Blacks. Nonetheless, even though adding the triglyceride levels in the regression model slightly increased the association between vitamin D and HOMA-IR (as assessed by standardized beta coefficients) in the whole studied sample, adding triglyceride levels in the model still did not make this association significant in the Non-Hispanic Blacks. Therefore, other factors can contribute more to the observed disparities in the Non-Hispanic Blacks. Various types of VDR genotypes and their related variants were related to the development of insulin resistance, which may potentially affect the individual response to vitamin D supplements (72, 73), and there probably could be racial disparities in the VDR polymorphism responsible for the lower association of vitamin D levels with HOMA-IR (21, 74–77). In addition, HOMA-IR mainly reflects hepatic insulin resistance, whereas vitamin D is more associated with insulin-mediated peripheral glucose uptake (78). Similarly, as for lower levels of serum triglycerides, intrahepatic fat, and intraperitoneal fat (70), it was shown that Black people have lower hepatic glucose production compared with other races/ethnicities, despite decreased whole-body insulin sensitivity and decreased peripheral (glucose disposal) and hepatic (suppression of glucose production) insulin sensitivity, compared with White people with the same body composition (79). Additionally, they have lower hepatic insulin clearance and increased insulin production, which probably could lead to increased insulin resistance, since chronic hyperinsulinemia can lead to insulin receptor desensitization (80, 81). Therefore, studies that include hyperinsulinemic-euglycemic clamp in the Non-Hispanic Blacks are needed to test if they will reveal different results compared with our study.

This study also found that Mexican Americans were more prone to be resistant to insulin, while non-Hispanic Whites were less susceptible. Mexican Americans have higher blood glucose levels and a greater family history of obesity, diabetes, and insulin resistance compared with the Non-Hispanic Whites (82). Mexican Americans with higher insulin levels were more likely to develop T2DM about 3–5 times more than the non-Hispanic Whites (82). A study about the genetics of variation in Mexican Americans demonstrated the importance of identifying HOMA-IR linkage on chromosome 12q24, as this region contained multiple candidate genes associated with obesity and diabetes (83).

This study has some potential limitations. The inherent properties of cross-sectional designs did not allow for verifying the causal relationships between vitamin D and insulin resistance. The study was limited to the non-diabetic population in the US, and the results cannot be extrapolated to the world; hence, larger multicenter analyses included would be more universally applicable. Additionally, HOMA-IR is only a substitute for a gold standard–a hyperinsulinemic-euglycemic clamp. The strengths are that we controlled for possible confounders and that we used a large scale and representative sample with precise super high-ultra performance liquid chromatography-tandem mass spectrometry for measurements of vitamin D serum levels.

In conclusion, race/ethnicity affected the negative association of vitamin D with insulin resistance assessed by HOMA-IR among the USA non-diabetic adults, as the negative association was not seen among the Non-Hispanic Blacks. While additional studies are required to verify the results of this study and explain the racial disparities, monitoring serum 25(OH)D may be useful in detecting those with vitamin D deficiency, starting with timely and adequate supplementation to prevent possible negative metabolic consequences.

## Data availability statement

Publicly available datasets were analyzed in this study. This data can be found here: https://wwwn.cdc.gov/nchs/nhanes/Default.aspx.

## Ethics statement

The studies involving human participants were reviewed and approved by Ethics Review Board of the National Center for Health Statistics Research (https://www.cdc.gov/nchs/nhanes/irba98.htm). The patients/participants provided their written informed consent to participate in this study.

## Author contributions

XY: conceptualization, methodology, formal analysis, investigation, and data curation, writing—original draft, writing—review and editing, visualization, supervision, and project administration. J-YC: formal analysis, software, validation, investigation, visualization, and writing—original draft. X-JH: programming, data curation, and writing—review and editing. JH-L, CH, WY, and N-XL: writing—original draft and writing—review and editing. W-CH: conceptualization. X-GG: project design and administration. All authors contributed to the article and approved the submitted version.

## Conflict of interest

The authors declare that the research was conducted in the absence of any commercial or financial relationships that could be construed as a potential conflict of interest.

## Publisher's note

All claims expressed in this article are solely those of the authors and do not necessarily represent those of their affiliated organizations, or those of the publisher, the editors and the reviewers. Any product that may be evaluated in this article, or claim that may be made by its manufacturer, is not guaranteed or endorsed by the publisher.

## Abbreviations

AA: associate of arts
ANOVA: analysis of variance
BMI: body mass index
CKD: chronic kidney disease
GED: general educational development
HOMA-IR: homeostasis model of insulin resistance
IQR: interquartile range
NHANES: National Health and Nutrition Examination Survey
PAL: physical activity level
PTH: parathyroid hormone
ROS: reactive oxygen species
SD: standard deviation
T2DM: type 2 diabetes mellitus
VDR: vitamin D receptor
VIF: variance inflation factor
25(OH)D: 25-hydroxyvitamin D.
