# Supplementary material for: Association between vitamin D serum levels and insulin resistance assessed by HOMA-IR among non-diabetic adults in the United States: Results from NHANES 2007–2014
Source: Front Nutr. 2022 Oct 14;9:883904. doi: 10.3389/fnut.2022.883904 (PMC9614433; doi:10.3389/fnut.2022.883904)
Supplement: Supplementary file 1 [file Data_Sheet_1.doc]

**Table S1. Standardized β of all covariates (predictors) in the fully adjusted model for prediction of log-transformed HOMA-IR.**

|  | **Standardized β** | **Unstandardized β (95%CI)** | **P** | **Collinearity statistics (VIF)** |
| --- | --- | --- | --- | --- |
| (Constant) |  | -0. 428 (-0.651, -0.205) | <0.001 |  |
| **Vitamin D** | -0.056 | -0. 001 (-0.001, 0.000) | <0.001 | 1.394 |
| **Age** | -0.080 | -0. 002 (-0.002, -0.001) | <0.001 | 1.634 |
| **Gender** | -0.021 | -0. 013 (-0.027, 0.000) | 0.056 | 1.284 |
| **Mexican American**^#^ | 0.071 | 0.062 (0.041, 0.082) | <0.001 | 1.476 |
| **Other Hispanics**^#^ | 0.042 | 0.043 (0.021, 0.064) | <0.001 | 1.205 |
| **Non-Hispanic Whites** (Reference) | - | - | - | - |
| **Non-Hispanic**^#^ **Blacks** | 0.021 | 0.017 (-0.002, 0.036) | 0.079 | 1.451 |
| **Other races**^#^ | 0.036 | 0.036 (0.015, 0.058) | 0.001 | 1.236 |
| **Education** | -0.004 | -0. 001 (-0.006, 0.004) | 0.717 | 1.259 |
| **BMI** | 0.477 | 0. 024 (0.023, 0.025) | <0.001 | 1.290 |
| **PAL score** | -0.078 | -0. 019 (-0.024, -0.014) | <0.001 | 1.166 |
| **Season of examination** | 0.001 | 0.001 (-0.012, 0.014) | 0.895 | 1.100 |
| **Current smoking** | -0.051 | -0.039 (-0.055, -0.023) | <0.001 | 1.137 |
| **Hypertension** | -0.010 | -0. 007 (-0.022, 0.008) | 0.367 | 1.183 |
| **Antihypertensive drugs** | -0.054 | -0. 045 (-0.064, -0.026) | <0.001 | 1.435 |
| **Sex hormones** | 0.021 | 0. 033 (0.002, 0.064) | 0.038 | 1.100 |
| **Statins** | -0.050 | -0. 054 (-0.078, -0.031) | <0.001 | 1.252 |
| **Serum bicarbonate** | -0.045 | -0. 007 (-0.010, -0.004) | <0.001 | 1.189 |
| **Serum triglycerides (log-transformed)** | 0.257 | 0. 330 (0.302, 0.357) | <0.001 | 1.226 |
| **Serum calcium** | 0.074 | 0. 282 (0.204, 0.361) | <0.001 | 1.150 |
| **Serum phosphorus** | -0.048 | -0. 087 (-0.124, -0.050) | <0.001 | 1.120 |

^**^P < 0.01

^*^P < 0.05

^#^Compared with Non-Hispanic Whites

Note: β = beta (regression coefficients); BMI = Body Mass Index; CI = confidence intervals; HOMA-IR = the homeostasis model of insulin resistance; PAL = Physical Activity Level; VIFs = Variance Inflation Factors.

**Table S2. Standardized β of all covariates in the association between serum vitamin D and log-transformed HOMA-IR** **in stratified races (fully adjusted model**).

|  | **Mexican American** | **Other Hispanic** | **Non-Hispanic White** | **Non-Hispanic Black** | **Other races** |
| --- | --- | --- | --- | --- | --- |
| **Serum vitamin D** | -0.073^**^ | -0.068^*^ | -0.034^*^ | -0.031 | -0.116^**^ |
| **Age** | -0.110^**^ | -0.052 | -0.058^**^ | -0.102^**^ | -0.105^*^ |
| **Gender** | -0.021 | 0.000 | -0.049^**^ | 0.044 | -0.043 |
| **Education** | -0.021 | 0.025 | -0.038^*^ | 0.035 | 0.02 |
| **BMI** | **0.472^**^** | **0.442^**^** | **0.481^**^** | **0.447^**^** | **0.511^**^** |
| **PAL score** | -0.062^*^ | -0.090^**^ | -0.064^**^ | -0.104^**^ | -0.080^*^ |
| **Season of examination** | -0.004 | 0.060 | -0.039^**^ | 0.089^**^ | -0.033 |
| **Current smoking** | -0.029 | -0.017 | -0.049^**^ | -0.097^**^ | -0.040 |
| **Hypertension** | -0.030 | -0.020 | 0.004 | -0.031 | -0.030 |
| **Antihypertensive drugs** | -0.047 | -0.002 | -0.060^**^ | -0.061^*^ | -0.069 |
| **Sex hormones** | 0.032 | 0.030 | 0.023 | 0.013 | -0.021 |
| **Statins** | -0.058^*^ | -0.101^**^ | -0.047^**^ | -0.042 | -0.016 |
| **Serum bicarbonate** | -0.047 | -0.058 | -0.033^*^ | -0.059^*^ | -0.064 |
| **Serum triglycerides (log-transformed)** | 0.322^**^ | 0.266^**^ | 0.238^**^ | 0.246^**^ | 0.228^**^ |
| **Serum calcium** | 0.060^*^ | 0.049 | 0.069^**^ | 0.117^**^ | 0.065^*^ |
| **Serum phosphorus** | -0.055^*^ | -0.027 | -0.051^**^ | -0.082^**^ | 0.044 |

^**^P < 0.01

^*^P < 0.05

Note: β = beta (regression coefficients); BMI = Body Mass Index; HOMA-IR = the homeostasis model of insulin resistance; PAL = Physical Activity Level.
